# *Ex Vivo* SIV-Specific CD8 T Cell Responses in Heterozygous Animals Are Primarily Directed against Peptides Presented by a Single MHC Haplotype

**DOI:** 10.1371/journal.pone.0043690

**Published:** 2012-08-22

**Authors:** Justin M. Greene, Emily N. Chin, Melisa L. Budde, Jennifer J. Lhost, Paul J. Hines, Benjamin J. Burwitz, Karl W. Broman, Jennifer E. Nelson, Thomas C. Friedrich, David H. O’Connor

**Affiliations:** 1 Department of Pathology and Laboratory Medicine, University of Wisconsin-Madison, Madison, Wisconsin, United States of America; 2 Department of Cellular and Molecular Biology, University of Wisconsin-Madison, Madison, Wisconsin, United States of America; 3 Wisconsin National Primate Research Center, University of Wisconsin-Madison, Madison, Wisconsin, United States of America; 4 Department of Biostatistics and Medical Informatics, University of Wisconsin-Madison, Madison, Wisconsin, United States of America; 5 Department of Pathobiological Sciences, University of Wisconsin, Madison, Wisconsin, United States of America; University of Pittsburgh Center for Vaccine Research, United States of America

## Abstract

The presence of certain MHC class I alleles is correlated with remarkable control of HIV and SIV, indicating that specific CD8 T cell responses can effectively reduce viral replication. It remains unclear whether epitopic breadth is an important feature of this control. Previous studies have suggested that individuals heterozygous at the MHC class I loci survive longer and/or progress more slowly than those who are homozygous at these loci, perhaps due to increased breadth of the CD8 T cell response. We used Mauritian cynomolgus macaques with defined MHC haplotypes and viral inhibition assays to directly compare CD8 T cell efficacy in MHC-heterozygous and homozygous individuals. Surprisingly, we found that cells from heterozygotes suppress viral replication most effectively on target cells from animals homozygous for only one of two potential haplotypes. The same heterozygous effector cells did not effectively inhibit viral replication as effectively on the target cells homozygous for the other haplotype. These results indicate that the greater potential breadth of CD8 T cell responses present in heterozygous animals does not necessarily lead to greater antiviral efficacy and suggest that SIV-specific CD8 T cell responses in heterozygous animals have a skewed focus toward epitopes restricted by a single haplotype.

## Introduction

Human immunodeficiency virus (HIV) continues to infect over 2.5 million individuals every year. Antiretroviral drugs successfully control viral replication in adherent individuals, but remain a treatment and not a cure for this disease. Recent successes in prevention, like that of the CAPRISA-004 microbicide trial and the Thai vaccine trial, demonstrate that several methods can reduce HIV incidence, but these trials also reveal the need to understand HIV pathogenesis and the correlates of protection in order to develop a more effective prophylactic vaccine [1], [2]. HIV vaccine design might benefit from a basic understanding of the immune components critical to mediating viral control. We do not currently know which components are necessary or sufficient to prevent HIV infection or ameliorate pathogenesis after infection.

Several avenues of HIV and simian immunodeficiency virus (SIV) research indicate that CD8 T cells play an important role in controlling viral replication during HIV infection. First, plasma viral loads decline within the first weeks of infection concordant with the rise in number of blood CD8 T cells [3]–[7]. Second, antibody mediated depletion of CD8 T cells leads to a simultaneous rise in plasma viral loads followed by viral load reductions with CD8 T cell recrudescence [8]–[14]. Finally, expression of certain major histocompatibility complex (MHC) class I alleles is known to influence virus load, survivorship, and/or rates of disease progression [15]–[17]. MHC class I proteins present endogenously derived peptides on the cell surface to surveilling CD8 T cells. Although the link between MHC and disease progression implies that particular CD8 T cell responses are involved in controlling virus replication, the reason why certain MHC class I molecules are associated with control of HIV and SIV is not clear.

In macaques, Mamu-B*17 and Mamu-B*08 correlate with control of viral replication and delayed progression to AIDS [18]–[19]. In humans, HLA-B*27 and HLA-B*57 expression is correlated with control of viral loads, while HLA-B*35 expression is correlated with rapid AIDS development [17], [20], [21]. Complicating this picture is the fact that not all individuals with protective alleles control viral replication. It remains unknown why only certain individuals with elite controller alleles maintain control of their viral loads [22].

These findings suggest that studying one allele in isolation, without considering the greater context of an individual’s MHC repertoire, will provide only a small piece of the total picture. Previous studies in mice examined MHC class I expression in MHC homozygous mice and their progeny. These studies demonstrated that alleles in the heterozygous F1 progeny of homozygous parents were expressed at rates different than 50% of that in the parental strain. This demonstrates that expression levels of a given allele can be variable depending on other alleles that are present. Moreover, transgenic expression of MHC class I alleles led to reduced CD8 T cell responses restricted by other MHC genes [23], [24]. These results suggest that mounting a certain CD8 T cell response is dependent on both having the restricting allele and the broader context of that alleles expression given the MHC allele repertoire. These MHC expression differences may affect the epitopic breadth of the CD8 T cell response.

The epitopic breadth of the CD8 T cell response may be an essential feature that determines whether viral load control can be established. Recognizing a broad array of HIV epitopes could, like multi-drug treatment, potentially reduce the chances that the virus can escape any single response. Reports assessing the contribution of breadth to HIV control are conflicting [25], [26]. One of the strongest demonstrations that CD8 T cell breadth may be critical to HIV control is heterozygote advantage. People or animals that are heterozygous for their MHC genes do better than than individuals that are homozygous. Heterozygote advantage is known to play a role in HIV, SIV, and several other pathogens [21], [27]–[29]. In studies of HIV, it has not been entirely clear whether this phenomenon is over-dominant or dominant. That is, it has not been demonstrated whether heterozygous individuals do as well as an individual homozygous for the more resistant haplotype (dominant) or better than an individual homozygous for the more resistant haplotype (over-dominant) [30]. If overdominant, it would suggest that the greater breadth of the CD8 T cell response is leading to improved outcomes.

Recently we examined Mauritian cynomolgus macaques containing known MHC haplotypes to assess the role of MHC-heterozygosity and epitopic breadth in controlling SIV replication. There were three groups of MHC-matched animals containing the M1 haplotype (M1/M1) the M3 haplotype (M3/M3) or both (M1/M3). These animals provided the unique opportunity to control for all MHC class I and II alleles unlike studies examining Mamu-B*17 or Mamu-B*08. We examined the CD8 T cell response in these animals by interferon gamma enzyme linked immunospot (IFNγ ELISpot), sequencing, and tetramer analysis. This research identified a single CD8 T cell response, restricted by an allele on the M1 haplotype that correlated with SIV control but did not identify a correlation between breadth and viral loads. Heterozygotes seemingly did not mount responses of greater breadth. Nevertheless, these were indirect measures of CD8 T cell function that may not adequately or directly assess CD8 T cell effectiveness. Here, we found that CD8 T cells from heterozygous animals inhibit viral replication most effectively on target cells bearing MHC class I molecules from only one of two potential haplotypes. If animals mounted effective CD8 T cell responses restricted by MHC class I alleles on both haplotypes, as suggested by the heterozygote advantage hypothesis, then one would expect the greatest suppression on matching heterozygous target cells which was not observed. These results provide the first evidence that, in this model, heterozygous macaques mount functional CD8 T cell responses restricted primarily by MHC class I alleles on one haplotype, suggesting that heterozygote advantage may be dominant but not overdominant.

## Methods

### Ethics Statement

All animals were cared for by staff at the Wisconsin National Primate Research Center (WNPRC) according to protocols approved by the Animal Care and Use Committee of the Graduate School of the University of Wisconsin-Madison.

Per AWA regulations, each animal was housed in an enclosure with at least 4.3, 6.0, or 8.0 sq. ft. of floor space, measuring 30, 32, or 36 inches high, and containing a tubular PVC or stainless steel perch. Each individual enclosure was equipped with a horizontal or vertical sliding door, an automatic water lixit, and a stainless steel feed hopper.

The nutritional plan utilized by the WNPRC for its nonhuman primate colony is based on recommendations published by the National Research Council. Twice daily the macaques on the described studies were fed a fixed formula, extruded dry diet (2050 Teklad Global 20% Protein Primate Diet) with adequate carbohydrate, energy, fat, fiber (10%), mineral, protein, and vitamin content. Feeding strategies were individually tailored to the age and physical condition of the experimental subjects. The dry diet was supplemented with fruits, vegetables, and other edible objects (e.g., nuts, cereals, seed mixtures, yogurt, peanut butter, popcorn, marshmallows, etc.) to provide variety to the diet and to inspire species-specific behaviors such as foraging.

The Behavioral Management Unit of the WNPRC provided foraging opportunities, food enrichment, human-to-monkey interaction, structural enrichment, and manipulanda to the experimental subjects to promote species-typical behavior and psychological well-being. Environmental enrichment objects were selected to minimize chances of pathogen transmission from one animal to another and from animals to care staff.

At least twice daily animals were evaluated for signs of pain, distress, and illness by observing appetite, stool quality, activity level, physical condition, etc. by staff at the WNPRC. If any of the above parameters were found to be abnormal, a member of the WNPRC veterinary staff was notified and appropriate clinical care was provided. Animals were euthanized on the recommendation of a clinical WNPRC veterinarian, if the veterinarian believed that the animal had developed an untreatable or incurable condition that caused significant pain or distress. Several SIV disease progression factors were also taken into consideration (e.g., inappetance, weight loss, opportunistic infection, etc.). Animals were euthanized by an intravenous (IV) overdose of sodium pentobarbital or equivalent as approved by a clinical veterinarian, preceded by ketamine. Some animals, required a final large blood draw be performed following anesthesia but prior to the IV overdose resulting in death by exsanguination. All euthanasia procedures were in compliance with the American Veterinary Medical Association’s Guidelines on Euthanasia.

### Animals and Infections

In the current study MCMs were intrarectally infected with 7,000 TCID50 molecularly cloned SIVmac239 Nef open virus. A panel of microsatellite markers spanning the MHC region was used to determine the haplotype of the MCMs as previously described by Wiseman et al [31]. At the time of this study, animals cy0321, cy0322, cy0326, cy0327, cy0332 and cy0333 were 43 weeks post-infection; animals cy0320, cy0324, cy0328, cy0329, cy0334 and cy0335 were 36 weeks post-infection; and animals cy0323, cy0325, cy0330, cy0331, cy0336 and cy0337 were 21 weeks post-infection.

### Plasma Viral Load Assay

We quantitated SIVmac239 plasma viral loads primarily using previously described techniques [28], [32], [33]. Briefly, viral RNA (vRNA) was reverse-transcribed and quantified using a LightCycler 2.0 (Roche, Indianapolis, IN). Serial dilutions of an SIV gag in vitro transcript were used for an internal standard curve. The limit of detection was set at 50 vRNA copies per ml of plasma.

### Viral Suppression Assay

On day one, PBMC were isolated from whole blood containing EDTA using Ficoll-Paque Plus (GE Healthcare Biioscience, Uppsala, Sweden) and density centrifugation. We depleted CD8 T cells from the PBMC using the Nonhuman primate CD8 microbead kit (Miltenyi, Auburn, Ca) and LS columns (Miltenyi) according to the manufacturer’s protocols. CD8-depleted target cells were resuspended in R15–50 (RPMI 1640 media (HyClone Laboratories, Logan, UT) supplemented with 15% fetal calf serum (Hyclone), 1% antibiotic-antimycotic, 1% l-glutamine (Hyclone), 50 units/ml IL-2 (NIH AIDS Research and Reference Reagent Program)) and 10 ug/ml Concanavalin A (ConA) at 1.5–2 million cells/ml in 37C with 5% CO_2_. On day 2, CD8 depleted target cells were washed with R10 (RPMI 1640 media supplemented with 10% fetal calf serum, 1% antibiotic-antimycotic, 1% l-glutamine) to remove ConA then resuspended in R15–50 and maintained at 37C with 5% CO_2_. On Day 4, target cells were harvested and counted on a Coulter Counter. These cells were resuspended at 2×10^6^/ml and plated in a 48 well plate. Cells were infected with SIVmac239 as previously described [32]. Target cells were washed once and resuspended in R15 at 2.5×10^5^ cells/ml and each target animal’s cells were plated in a separate 96-well round bottom plate (Corning, Corning, NY) at 2.5×10^4^/well. Outside wells were filled with 1×PBS (Hyclone) to prevent evaporation. PBMC were isolated from whole blood as previously described and CD8 T cells (effector cells) were enriched using CD8β-phycoerythrin (PE) antibody (Beckman Coulter, Fullerton, CA), anti-PE microbeads (Miltenyi) and LS columns (Miltenyi). Effector cells were added to the relevant wells on each 96 well plate. On day eight, cells were stained for intracellular p27 as described below.

### Flow Cytometry

On day eight, plates were centrifuged at 670 g for 5 minutes and media was aspirated from each plate. Each well was stained with CD8 Pacific Blue (BD Biosciences, San Jose, CA), CD4 PE Cy7 (BD Biosciences), CD3 Alexa Fluor 700 (A700) (BD Biosciences), W6/32 allophycocyanin (APC) (NIH AIDS Research and Reference Reagent Program; kindly provided by David Watkins) for 30 minutes. Cells were washed twice and then fixed with 2% paraformaldehyde. After twenty minutes, cells were washed twice with FACS buffer (1×PBS (Hyclone) supplemented with 10% FBS (Hyclone)). Cells were then stained intracellularly with anti-Gag p27 fluorescein isothiocyanate (FITC) antibody 55–2F12 (NIH AIDS Research and Reference Reagent Program; kindly provided by David Watkins) in 100uls of Permeabilization Medium (Invitrogen, Carlsbad, CA). These cells were then washed twice with FACS Buffer and fixed with 2% PFA. We analyzed the samples using the BDLSRII and the HTS (BD Biosciences). Final analysis was performed using Flowjo 9.3.2 (Treestar, Ashland, OR). The percentage of infected targets was determined by gating on lymphocytes using forward and side scatter, then gating on CD8- cells, then gating out autofluorescent cells, and finally gating on the number of p27+ cells present in this population.

### Statistical Analysis

The normalized percent suppression was calculated using the following formula: (%p27+ cells in no effector wells - %p27+ cells in test well) / (%p27+ cells in no effector well)×100. In these plots, viral suppression on target cells from animal cy0335 was not included because thirteen of eighteen animals were unable to suppress viral replication above 15% on this animal’s target cells. We used a one-way ANOVA with Tukey’s Multiple Comparison post-test to compare the columns in individual graphs with Prism (GraphPad Software, La Jolla, CA). We used Prism to assess whether there was a correlation in XY-plots comparing viral loads against another variable.

## Results

### Animal Identification and Viral Inhibition Assay Development

We used eighteen SIVmac239 infected MCM with one of three genetic backgrounds identified by microsatellite typing [31]. This group of MCM included six animals homozygous for the M1 MHC haplotype (M1/M1), six animals homozygous for the M3 MHC haplotype (M3/M3) and six animals heterozygous for the M1 and M3 MHC haplotypes (M1/M3). The M3 haplotype has previously been associated with control of SHIVsbg *in vivo* [34]. This haplotype does not seem to correlate with spontaneous virologic control of SIVmac239; however, fifty percent of animals with the M1 haplotype naturally control SIVmac239 replication [28], [35].

**Figure 1:**
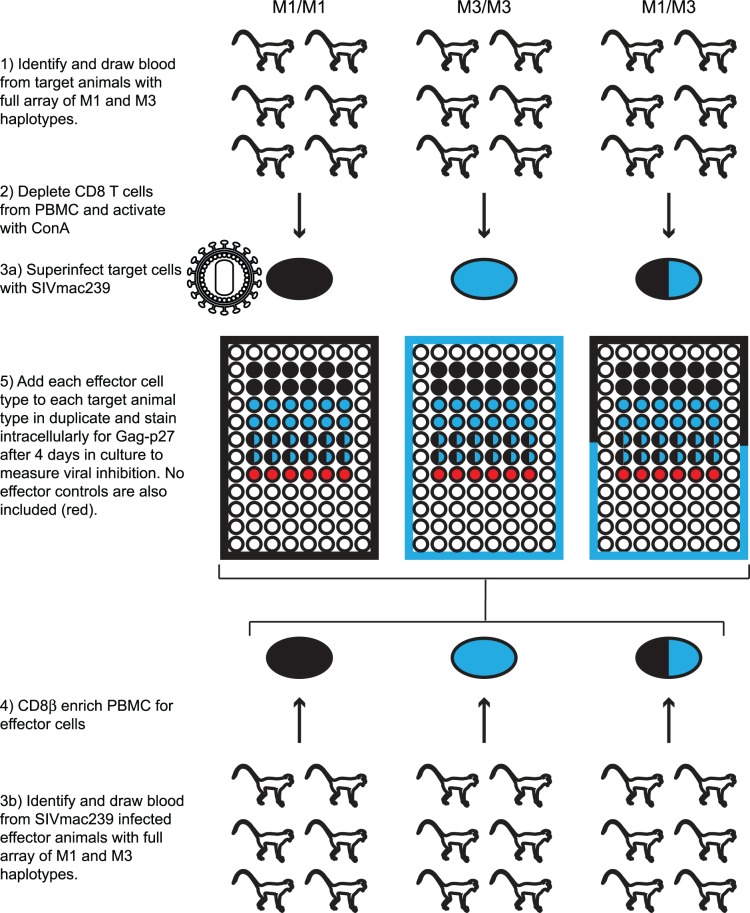
Viral suppression assay schematic. Target cells are prepared on Day 1 by CD8 depleting PBMC and stimulating cells with ConA. Cells are washed on Day 2. On Day 4 target cells are superinfected *in vitro* with SIVmac239 and plated in a 96 well plate. Effector cells are prepared by isolating CD8β T cells from PBMC. Effector cells are added to target cells in a 1∶1 ratio. Each combination of effector and target cells was compared in this assay.

We CD8 depleted PBMC from all eighteen animals on day 1 and stimulated the cells with ConA to create target cells. On Day 4 we CD8β enriched PBMC to obtain CD8 T cells for effector cells. We infected the target cells *in vitro* with SIVmac239 using the previously described magnetofection technique [32], [36]. We then added effector cells from all eighteen animals to target cells from the same eighteen animals and intracellular stained the samples for Gag-p27 on day 8. We performed all 324 possible combinations of effector and target animals (Fig. 1). We also included wells with infected target cells but no effector cells to measure the maximum level of viral replication. We determined the normalized percent suppression using the following equation: (Average %p27+ cells in the no effector wells – Average %p27+ cells in the test wells)/ Average %p27+ cells in the no effector wells.

**Figure 2:**
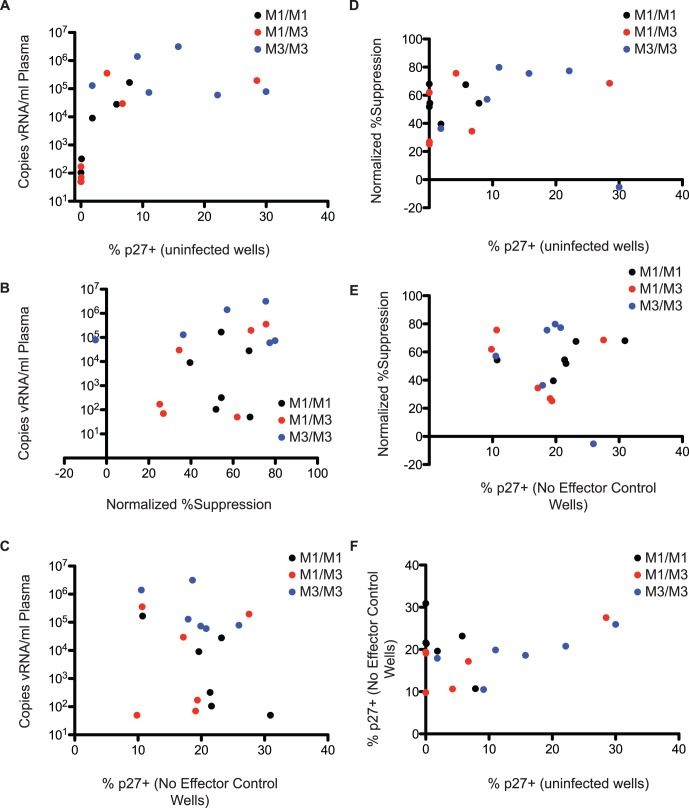
Viral load does not correlate with CD8 T cell efficacy in vitro. A) Plasma viral loads were plotted against the percentage of p27+ cells after eight days in culture in wells where cells were not superinfected in vitro. These results display the growth of autologous virus. B) Viral loads were plotted against the normalized percent suppression on autologous target cells. C) Viral loads were plotted against the percentage of target cells that were p27+ after in vitro superinfection in the no effector control wells. D) The normalized % suppresion was plotted against the percentage of p27+ targets in the no infection control wells. E) Normalized percent suppression was plotted against the percent of p27+ targets in the no effector control wells. F) The percent of p27+ targets in the no infection wells was plotted against the percent of p27+ targets in the no effector control wells.

We used fresh cells from infected animals to generate target cells for the assay because we were unable to expand previously frozen cells in sufficient numbers. We plated cells that were not superinfected *in vitro* from all animals to assess whether high plasma viral loads would lead to growth of cell-associated virus. We found that, at viral loads above 1×10^4^ copies of vRNA per ml plasma, we could measure growth of autologous virus from some animals (Fig. 2A).

### Suppression of Viral Replication *in vivo* did not Correlate with Suppression of Viral Replication *ex vivo*

We compared the ability of CD8 T cells to suppress viral replication on autologous target cells against viral loads in the animals. We expected that CD8 T cells isolated from animals maintaining low plasma viral loads would suppress viral replication most effectively *ex vivo*. Surprisingly, we found that there was no correlation between plasma viral loads and the ability for bulk CD8 T cells to suppress viral replication on autologous target cells (Fig. 2B). It is entirely possible that multiple mechanisms are responsible for control *in vivo*.

We also examined whether plasma viral loads correlated with the ability to infect target cells *in vitro*. There was no correlation between the frequency of p27+ targets at the completion of the assay in no effector control wells and the plasma viral loads *in vivo* (Fig. 2C). Cells from elite controllers are equally susceptible to infection *in vitro* as measured by this assay. These results indicate that there is not an inherent quality of the CD4 T cells in MCM controllers that makes them refractory to infection. Viral suppression did not correlate with the level of viral replication in the no infection control wells or no effector control wells (Fig. 2D and E). Additionally, there was no correlation between the percentage of p27+ targets in the no infection control wells and the no effector control wells (Fig. 2F).

### Homozygous Animals Suppressed Viral Replication Poorly on Mismatched Target Cells

Next we examined the ability of CD8 T cells from M1/M1 and M3/M3 animals to suppress viral replication on target cells from animals with all three genetic backgrounds. Given the potential problems in this assay arising from the use of allogeneic target and effector cells, we assessed whether there were obvious differences in the ability to suppress viral replication on autologous target cells compared to allogenic MHC-matched target cells. We found that, in general, there was a heterogeneous ability to suppress viral replication on MHC class I and class II matched, non-autologous target cells (Table S1). We found that in 51 of the 90 possible allogenic MHC-matched combinations, effector cells suppressed more effectively on autologous target cells. These results indicate that, while there may have been slightly higher levels of suppression on autologous target cells, the use of non-autologous target cells did not have a large effect on viral suppression, and the assay could be used to compare viral suppression by an effector animal on target cells from different animals. Certain target animals were more or less sensitive to viral suppression but this was not correlated with *in vivo* viral loads or the percentage of p27+ cells in the no-effector control wells (Data not shown).

**Figure 3:**
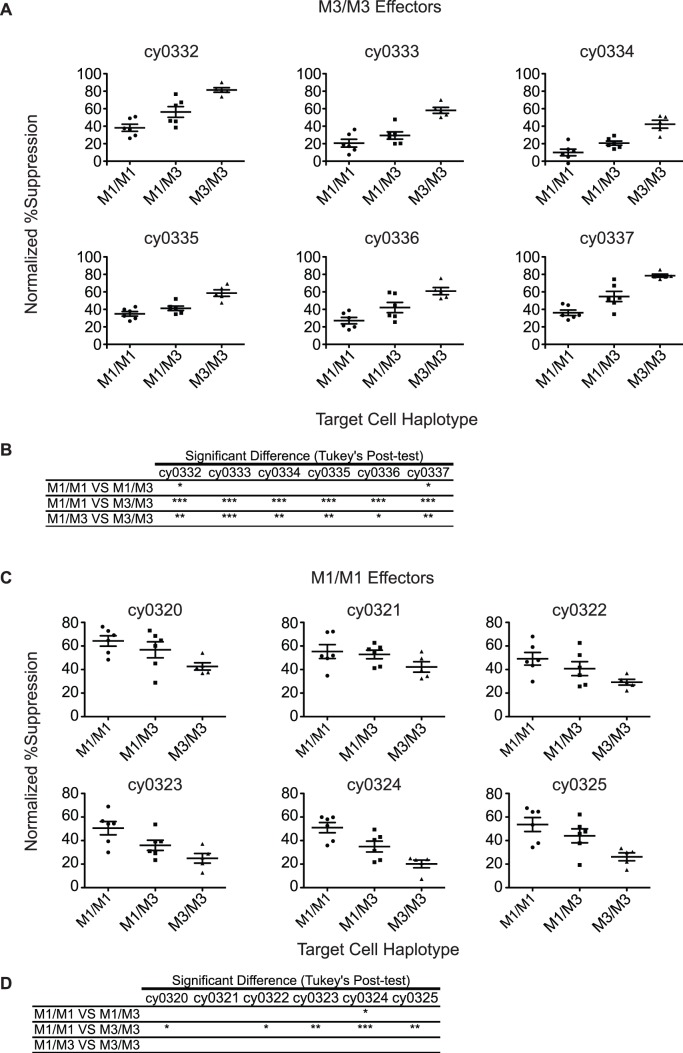
Viral suppression by effector cells from homozygous animals. A) Each plot represents the results from an individual M3/M3 effector animal on each target animal. Each column in the graph displays a different target animal haplotype. B) Table showing the Tukey’s Multiple Comparison Test of the columns in each of the graphs (*p<0.05; **p<0.01; ***p<0.001). C) Each plot represents the results from an individual M1/M1 effector animal on each target animal. Each column in the graph displays a different target animal haplotype. D) Table showing the Tukey’s Multiple Comparison Test of the columns in each of the graphs (*p<0.05; **p<0.01; ***p<0.001).

CD8 T cells from M3/M3 animals suppressed viral replication more effectively on M3/M3 target cells than on M1/M3 target cells (Fig. 3A and B). In contrast, except for animal cy0324, M1/M1 animals suppressed viral replication well on both M1/M1 and M1/M3 target cells (Fig. 3C and D). As expected, both M1/M1 and M3/M3 effector cells suppressed viral replication less effectively on mismatched homozygous target cells. The M1 and M3 haplotypes share two common Mafa-A alleles that are known to restrict CD8 T cell responses, and this may account for the low levels of inhibition of viral replication on homozygous mismatched target cells [35], [37], [38]. The fact that M3/M3 effector cells do not suppress viral replication as well on M1/M3 target cells as they do on M3/M3 target cells indicates that M1/M3 target cells may exhibit differences in antigen processing and/or presentation of peptides bound to M3 MHC molecules compared to M1 MHC moelecules. These differences may lead to lower levels of SIV peptide-loaded M3 MHC proteins on the cell surface and to impaired target cell recognition by CD8 T cells from M3 animals.

### CD8 T Cells from M1/M3 Animals Suppress Viral Replication Equally Well on M1/M1 and M1/M3 Target Cells

**Figure 4:**
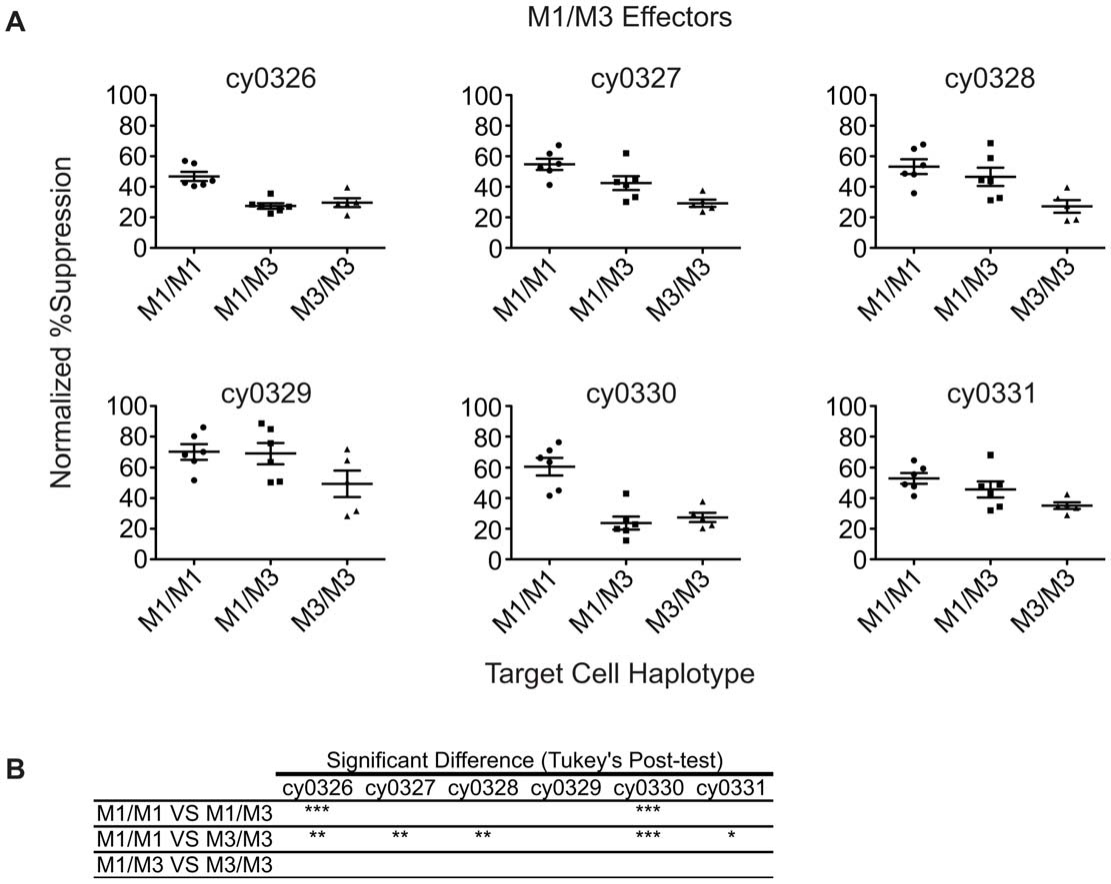
Viral suppression by effector cells from heterozygous animals. A) Each plot represents the results from an individual M1/M3 effector animal on each target animal. Each column in the graph displays a different target animal haplotype. B) Table showing the Tukey’s Multiple Comparison Test of the columns in each of the graphs (*p<0.05; **p<0.01; ***p<0.001).

We combined CD8 T cells from M1/M3 animals with target cells from M1/M1, M3/M3 and M1/M3 animals. We noted very poor suppression of viral replication on target cells of the M3/M3 background (Fig. 4A and B). We found that M1/M3 CD8 T cells reduced viral replication by roughly 50% on both M1/M1 and M1/M3 target cells. Surprisingly CD8 T cells from M1/M3 animals did not suppress viral replication most effectively on matched target cells. Instead, effectors from M1/M3 animals suppressed viral replication most effectively on M1/M1 target cells. Thus, functional CD8 T cells in heterozygous macaques predominantly target SIV-peptides restricted by the M1 haplotype.

## Discussion

CD8 T cells are a critical component of the immune response against intracellular pathogens. In HIV infection, CD8 T cells help to contain viral replication after infection but do not completely clear infected cells. It is possible that if these CD8 T cell responses were logged into immunological memory after vaccination and present at the time of infection, they could prevent transmission, prevent the rapid dissemination of virus to tissue reservoirs or permanently control virus replication. Thus far, we have not defined the required quality, quantity, or focus of a CD8 T cell response that controls viral replication. As an important side-note, it is also unclear whether the responses that control viral replication after infection are the same responses that prevent HIV acquisition.

We used groups of completely MHC-matched MCM to study the CD8 T cell response while minimizing the confounding effects of host genetic diversity. We used eighteen animals with defined MHC haplotypes and performed 324 different E:T combinations. Specifically, we included animals that were M1/M1 homozygous, M3/M3 homozygous, and M1/M3 heterozygous to explore how MHC class I genes on one haplotype can affect the CD8 T cell responses restricted by the other haplotype. We also used this model to explore the breadth of the CD8 T cell response in heterozygous animals. Unlike tetramer stains that strictly examine the frequency of SIV-specific CD8 T cells or assays that use indirect assessments of CD8 T cell function through measuring cytokine production, viral suppression assays directly measure the ability of CD8 T cells to prevent virus replication. Using this assay, we were able to examine whether CD8 T cells from heterozygous animals suppress viral replication on target cells bearing zero, one or two matched MHC class I haplotypes. Better suppression on multiple haplotypes may indicate that animals have greater breadth of CD8 T cells that suppress viral replication or greater functional breadth.

We expected M1/M3 effector cells to recognize a greater variety of epitopes on M1/M3 target cells than on M1/M1 or M3/M3 target cells leading to greater suppression of viral replication on M1/M3 target cells. However, M1/M3 effector cells did not suppress viral replication most effectively on M1/M3 target cells. These results indicate that heterozygous animals do not mount a CD8 T cell response of greater functional breadth than homozygous animals. Moreover, we found that M1/M3 effector cells frequently suppressed viral replication most effectively on M1/M1 target cells suggesting that suppressive responses in these heterozygous animals were primarily directed against epitopes restricted by alleles on the M1 haplotype and that the contribution of the M3 responses is small. One explanation for these results is that M1/M1 animals are presenting a greater amount of M1-restricted peptides on the surface of their cells in comparison to M1/M3 target cells, facilitating recognition by CD8 T cells restricted by alleles encoded on the M1 haplotype.

The differences we observed in effector cell ability to suppress viral replication on M1/M3 target cells compared to matched target cells is particularly interesting. M3/M3 animals suppress viral replication significantly better on matched target cells than they do on heterozygous target cells. This is different from the M1/M1 animals that suppress viral replication well on MHC matched and heterozygous target cells. Combined, these findings indicate that there may be reduced presentation of M3 restricted epitopes on the surface of M1/M3 cells. These results mirror those from studies on other pathogens including HIV, CMV and EBV [39]–[41]. Immunodominance is a multifaceted phenomenon and there are likely several mechanisms responsible for our observations. These may include the availability of antigenic peptides, the expression of different MHC class I alleles and the precursor frequencies of SIV-specific CD8 T cells [42]–[44]. Ultimately, these results suggest that heterozygous animals will mount functional CD8 T cell responses of limited focus despite the potential capacity for infected cells to present a greater variety of peptides on their surface.

The apparent dominance of M1-restricted CD8 T cell responses in M1/M3 animals and M1/M1 animals, in addition to the observed correlation between the M1 haplotype and control of viral loads, suggests that these responses may have a protective role during SIV infection. While we were unable to correlate viral inhibition with viral loads, this may, in part, be due to the outgrowth of autologous virus in our assay. Nevertheless, the lower viral loads in M1+ animals indicate that CD8 T cells in these animals could be playing an essential role in the containment of viral replication [35]. Our results may also have important implications for vaccine studies in other models like the rhesus or pigtail macaques which contain known ‘protective’ MHC class I alleles [15], [16], [45]. Critically, specific responses restricted by these alleles may be responsible for control and vaccines targeted at generating responses of greater breadth may be better served by including minimal peptide antigens over whole protein constructs or focusing specifically on individual responses at the expense of epitopic breadth. At first glance, these findings appear negative for those with ‘non-protective’ MHC class I alleles, but it may be that protective responses in these individuals are subdominant. Vaccination for these subdominant responses may help non-controllers more effectively inhibit viral replication. Additionally, it is possible that, while our study does not indicate breadth is important to viral control, other models may provide different information or more nuanced views of the role of CD8 T cells in viral control.

Several additional factors might have played a role in the variable suppression we witnessed in this assay. First, the virus growing in target cells from animals with high viral loads may have contained CD8 T cell escape mutations. In a separate, but related study, we found that virus in M3/M3 animals escaped the HW8 CD8 T cell response which is correlated with *in vivo* control of virus replication. The virus escaped with slower kinetics in animals that control viral loads like the M1 animals. This may have lead to a reduced capacity to suppress viral replication by M1/M1 effector cells. We do not have sequence data from the assay to assess whether the frequency of sequence variants correlated with suppression. Additionally, it may be possible that CD4 or CD8 T cell dysfunction played a role in reduced suppression. Vaccinated macaques may exhibit a different capacity to suppress viral replication on targets containing different MHC-haplotypes. Finally, it is not clear whether the suppression we observed is entirely SIV-specific or if the CD8 cell antiviral factor (CAF) is playing a role in the suppression we are observing in this assay [46].

These experiments would have been impossible in humans or other outbred macaque species. These studies represent the first time that large groups of MHC-matched animals could be studied in the context of AIDS-virus infection. Using groups of six MHC-matched animals made it possible to perform studies *ex vivo* that reached statistical significance and combined for 324 different E:T combinations. They provide a provocative finding that, if true, demonstrates some haplotypes contain certain alleles that are dominant to others in SIV infection. Nevertheless, we were unable to perform these studies in animals containing alternative MHC-haplotypes. It is possible that this finding is unique to these two MHC-haplotypes and that if one performed this experiment with HIV infected individuals, heterozygote effector cells might suppress equally well on targets bearing either matched MHC-haplotype. VSAs with MCM provide a unique model for understanding the functional breadth and efficacy of CD8 T cells in the context of SIV infection. By using this assay with other viruses or vaccine strategies, the VSA in MCM may prove useful to evaluating future HIV vaccine candidates.

## Supporting Information

Table S1 **Comparison of viral suppression on matched targets to viral suppression on autologous targets.** Numbers in the table show the difference between the suppression on autologous targets compared to each MHC-matched animal (Normalized %Suppression on Matched animal - Normalized %Suppression Autologous). Negative numbers indicate tests where animals suppressed viral replication better on autologous targets. Positive numbers indicate tests where animals suppressed viral replication less effectively on autologous targets.
(EPS)
